# Disulfiram/Copper induces antitumor activity against gastric cancer via the ROS/MAPK and NPL4 pathways

**DOI:** 10.1080/21655979.2022.2038434

**Published:** 2022-03-15

**Authors:** Yao Liu, Xin Guan, Meiling Wang, Naixue Wang, Yutong Chen, Baolei Li, Zhuxuan Xu, Fangwei Fu, Cheng Du, Zhendong Zheng

**Affiliations:** a Department of Oncology, General Hospital of Northern Theater Command, Dalian Medical University, Shenyang, P. R. China; b Department of Oncology, Northeast International Hospital, Shenyang, P. R. China; c Department of Oncology, General Hospital of Northern Theater Command, Shenyang, P. R. China; d Department of Oncology, General Hospital of Northern Theater Command, Jinzhou Medical University, Shenyang, P. R. China; e Department of Oncology, General Hospital of Northern Theater Command, China Medical University, Shenyang, P. R. China; f Department of Oncology, General Hospital of Northern Theater Command, Shenyang Pharmaceutical University, Shenyang, P. R. China

**Keywords:** Disulfiram, gastric cancer, reactive oxygen species, ubiquitinated protein, nuclear protein localization protein 4

## Abstract

Disulfiram (DSF) is an anti-alcoholism medication with superior antitumor activity and clinical safety; its antitumor mechanisms in gastric cancer (GC) have not been fully explored. In the present work, low nontoxic concentrations of copper (Cu) ions substantially enhanced DSF’s antitumor activity, inhibiting the proliferation and growth of GC cell lines. DSF/Cu elevated the generation of reactive oxygen species (ROS), and apoptosis was induced in an ROS-dependent manner. This process might involve primary inhibition GC by DSF/Cu through induction of apoptosis via the ROS/mitogen-activated protein kinase pathway. Disordering transportation of ubiquitinated protein may also fuel the process. In summary, we found that DSF exerts antitumor effects on GC. DSF/Cu should be considered as adjunctive therapy for GC.

## Graphical Abstract

**Figure:**
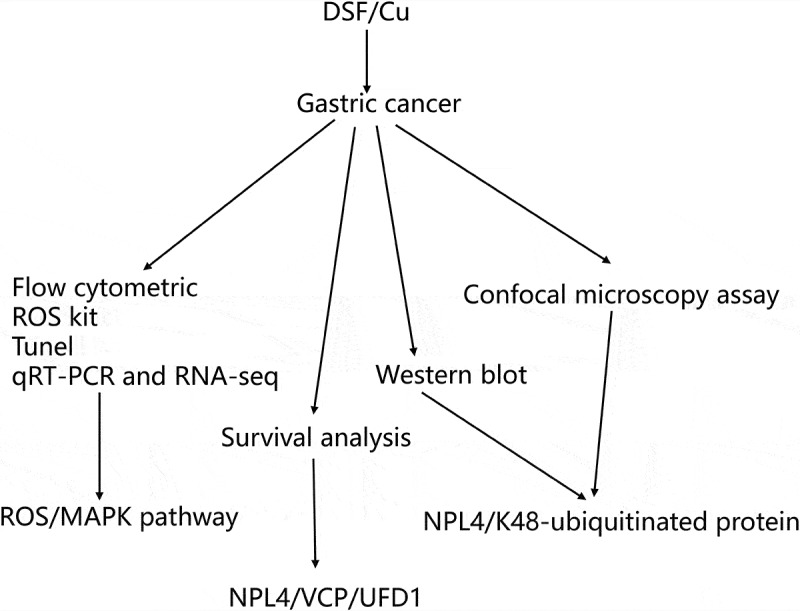

## 1. Introduction

Gastric cancer (GC) is a lethal malignancy, ranking fifth in incidence and fourth in mortality among all cancers in 2020 [1]. In addition to chemotherapy, treatments for advanced GC include immunotherapy and targeted therapy [2]. Nevertheless, only a minority of patients benefit from these treatments, necessitating new therapeutic agents.

Developing new medicines is a long and challenging process because of high costs, failure rates, and prolonged testing periods. Repositioning previously approved drugs as candidate therapeutics represents a faster and less expensive approach with higher success rates [3]. Many successful cases inspired investigators, including thalidomide [4] and aspirin [5]. Disulfiram (DSF) is a candidate drug with substantial potential to be repositioned; it is an anti-alcoholism drug approved by the US Food and Drug Administration in 1951 [6] that has been studied in various cancers because of its antitumor activity [7]. Several lines of evidence suggest the anticancer activity of DSF with several mechanisms focused on elevating intracellular reactive oxygen species (ROS) levels [8–10], reducing acetaldehyde dehydrogenase (ALDH) expression to weaken the stemness of cancer cells [8,11–13], and disturbing the ubiquitin-proteasome system [14–16], or causing DNA damage [17,18]. These antitumor effects were reported in many preclinical studies and recently in several types of human cancer (prostate [19], liver [20,21], and ovarian cancer [8], glioblastoma [17,22], lung [23], breast [24], pancreatic [24], acute myeloid leukemia [9], melanoma [25], and GC [26,27])

The present study explored molecular mechanisms by which DSF/copper (Cu) combats GC to suggest promising therapeutic targets. We used genomics via RNA-seq to investigate these. We hope these findings provide insights into the anti-GC mechanisms of DSF/Cu.

## 2. Materials and methods

### 2.1 Cell culture and drug preparation

SGC-7901 and HGC-27 were purchased from the Shanghai Collection Cell Bank of the Chinese Academy of Sciences. Cells were cultured in complete RPMI 1640 medium with 10% fetal bovine serum and 1% penicillin/streptomycin (HyClone, Logan, Utah, USA). Free DSF and Cu gluconate (Sigma-Aldrich, St. Louis, US) were dissolved in dimethyl sulfoxide (DMSO, Sigma-Aldrich, St. Louis, US) and stoked at a concentration of 100 mM at 4°C.

### 2.2 Cell cytotoxicity assay

As described previously, a CCK-8 assay was used for cell viability testing [15]. HGC-27 and SGC-7901 cells were cultured overnight in 96-well plates at 5 × 10^3^ cells per well. The following day, the cells were treated with DMSO, DSF (0.24 or 0.30 μM), Cu (0.2 μM), or DSF (0.24 μM or 0.30 μM) + Cu (0.2 μM) for 24 h. We added CCK-8 solution (10 μl) (Dojindo Molecular Technologies, Kumamoto, Japan) and incubated it with cells for 2 hours. A microplate reader measured the absorbance was measured at 450 nm using a microplate reader (Bio-TEK Instruments, USA).

### 2.3 Flow cytometric analysis and measurement of intracellular ROS accumulation

Cells were plated in 6-well plates at 1 × 10^4^ cells per well, as described previously [28]. After overnight incubation, cells were treated with various concentrations of DSF, Cu, DSF/Cu combinations for 2 hours. Cells without treatment were used as unstained controls. The intracellular ROS was determined by a fluorescent ROS detection kit (2.5 μM) (Yishen Biotechnology, Shanghai, China). Flow Jo 10.0 software and Image J 1.52 were used for data analysis.

### 2.4 Western blotting analysis

Western blotting was used to determine protein expression [29]. The logarithmic growth GC cells were treated with indicated concentrations for 24 hours and then lysed in a mixture of RIPA buffer and PMSF (1000:1), followed by protein purification. The protein samples (20 μg) were separated by 10% or 12% sodium dodecyl sulfate-polyacrylamide gel electrophoresis, transferred to nitrocellulose membranes, and blocked with 5% BSA for 2 h at 37°C. Membranes were incubated with primary antibodies (anti-NPL4,1:10,000, Abcam, cat. no: ab224435; anti-K48-ubiquitin 1:10,000, Abcam, cat.no:ab140601; anti-β-actin 1:5000, ABclone, cat. no: AF7018) overnight at 4°C followed by incubation with secondary antibodies for 2 h at room temperature. Finally, the membranes were incubated with an ECL reagent, and the images were captured using the Versa Doc imaging system (Bio-Rad, USA).

### 2.5 Confocal microscopy assay

The confocal microscopy assay was performed as described previously [14]. Cells were seeded on plastic inserts in 12-well dishes, then treated with the drugs for 24 hours, fixed with 4% formaldehyde at room temperature for 15 minutes, washed with phosphate-buffered saline (PBS) three times, and then permeabilized in 0.5% Triton X-100 for 20 minutes. Cells were washed with PBS three times and then blocked with 5% goat serum at 37°C for three minutes. This was followed by immunostaining with primary antibody (anti-NPL4, 1:10,000, Abcam, cat. no: ab224435; anti-K48-ubiquitin 1:10,000, Abcam, cat. no: ab140601) for 2 h at room temperature, washing with PBS three times, and staining with fluorescently-conjugated secondary antibody for 60 min at room temperature. The nuclei were stained with DAPI at room temperature for 2 min after PBS washes. The pieces were removed, sealed with a sealing solution containing an anti-fluorescence quenching agent, and observed using laser scanning confocal microscopy (Carl Zeiss, China).

### 2.6 qRT-PCR and RNA-seq

qRT-PCR and RNA-seq were performed as described previously [10]. HGC-27 and SGC-7901 cells were treated with DSF, Cu, or DSF/Cu for 24 h. Total RNA was extracted using TRIzol reagent, quantified using a spectrophotometer (Bio-Tek, CA, USA), and reverse transcribed into cDNA using Prime Script RT Master Mix Kit. Real-time PCR was performed using a SYBR Premix Ex Taq II Kit (Takara, Dalian, China). The relative fold-changes of mRNAs were calculated. Primers are listed in Table S1.

RNA sequencing was performed using Illumina HiSeq2500 (Gene Denovo Biotechnology, Guangzhou, China). Briefly, total RNA was extracted (three samples per group), enriched, and reverse transcripted into cDNA. Then, the cDNA fragments were purified, end-repaired, poly(A) added, and ligated to Illumina sequencing adapters. The ligation products were size-selected, PCR-amplified, and sequenced. Fastp (version 0.18.0) was used to assess the raw data quality. RNAs differential expression analysis was performed using DESeq2 software. P-value < 0.05 and absolute fold-change ≥ 1.5 were considered significant. The Gene Ontology database and Kyoto Encyclopedia of Genes and Genomes (KEGG) were used to analyze data.

### 2.7 TdT-mediated dUTP-biotin nick end labeling (TUNEL) staining

TUNEL staining was performed as described previously [16]. We fixed air-dried cell samples with freshly prepared 4% paraformaldehyde for 1 hour at 25°C, washed with PBS three times, and incubated for 2 minutes on ice (4°C). After PBS washes, we added 50 µl TUNEL reaction mixture (Roche, Sigma-Aldrich, cat. no: 12,156,792,910), incubating slides in a humidified atmosphere for 60 min at 37°C in the dark. Cells were rewashed in PBS, and fluorescence images were obtained using Nikon fluorescence microscopy (AMG, Botell, Washington, USA). Image J 1.52 and GraphPad Prism 8.0 software calculated the number of apoptotic cells.

### 2.8 Survival analysis using Kaplan-Meier plots

Kaplan-Meier (KM) survival curves were drawn for high- and low-expression groups to show prognostic values of NPL4, UFD1, and VCP in patients with GC based on the KM plotter database [30]. These data sets included GSE14210, GSE15459, GSE22377, GSE29272, GSE51105, and GSE62254. GSE62254 was excluded from the total sample survival analysis because of its markedly different clinical and genomic data, as suggested by the KM plotter. Survival data and expression levels were recorded. The best cutoff values were determined by algorithms embedded in the KM plotter. The final prognostic KM plots were presented with a log-rank *p-value*. P-values < 0.05 were considered significant.

### 2.9 Statistical analysis

All data presented were representative of three independent experiments. Statistical analyses were performed using IBM SPSS 26, GraphPad Prism 8.0, and FlowJo 10.0. The group data were expressed as the mean ± standard error. Differences were considered significant at P < 0.05.

## 3. Results

We investigated the mechanisms by which DSF and Cu exert anti-GC effects using RNA-seq and bioinformatic analysis and found that ROS/MAPK was involved in apoptosis-related cell death. NPL4 was a critical cofactor of P97 that plays a crucial role in ubiquitinated protein transporting, also associated with cell death.

### 3.1 DSF/Cu inhibited cell viability of GC cells

**Figure 1.**
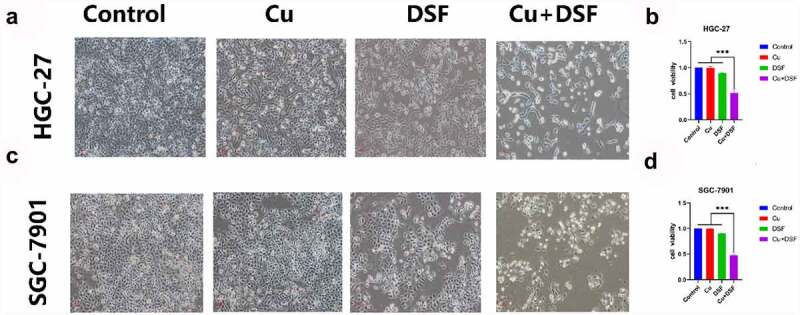
**DSF/Cu inhibits the viability of GC cells**. HGC-27 and SGC-7901 were exposed to Cu (0.2 μM), DSF (0.24 μM or 0.3 μM), or DSF + Cu (0.24 μM, 0.3 μM +0.2 μM) for 24 h. (a) The morphologic changes (X100 magnification) of HGC-27 after 24 h of drug exposure. (c) Cell viability of HGC-27 was analyzed across groups. (b) The morphologic changes (X100 magnification) of SGC-7901 after 24 h of drug exposure. (d) Cell viability of SGC-7901 was analyzed across groups at selected concentrations. Scale bar: 100 µm. Data are expressed as mean ± standard deviation of three independent experiments. * p < 0.05 vs. the DSF/Cu group.

We selected 0.2 μM as the Cu ion working concentration, with 0.24 μM for DSF for HGC-27 and 0.30 μM for the SGC-7901. DSF/Cu co-treatment inhibited cell viability of HGC-27 and SGC-7901 compared to DSF or Cu alone. Cell viability was 49% in DSF/Cu, 90% in DSF, and 100% in Cu (Figure 1). DSF/Cu-treated cells were shrunken, floating, and rounded, more than cells treated with DSF or Cu alone. This finding suggests that the nontoxic concentration of Cu significantly potentiated the cytotoxicity of DSF.

### 3.2 DSF/Cu induces cytotoxicity via modulating the expression of genes involved in MAPK signaling pathways

**Figure 2.**
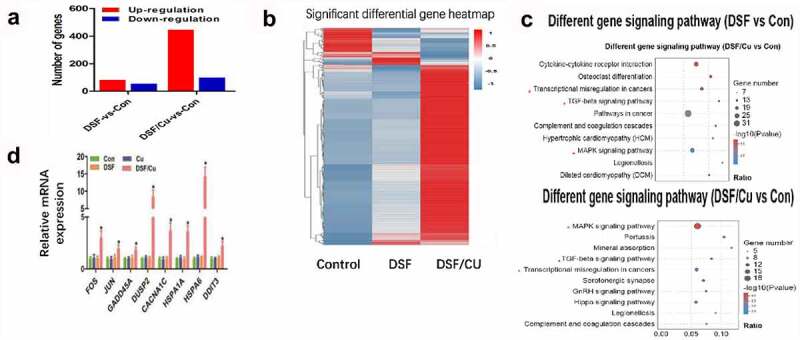
**DSF/Cu induced antitumor activity through MAPK pathway**. HGC-27 were exposure to DSF (0.24 μM), DSF + Cu (0.24 μM +0.2 μM) for 24 h. (a) Numbers of up- (red) or down-regulated (blue) genes in HGC-27 cells of different groups. (b) Heatmap showing expression fold change of genes in DSF or DSF/Cu treated cells compared to control group. (c) Pathway enrichment analysis of the differentially expressed genes based on the KEGG database. (d) The mRNA expression of genes involved in the MAPK signaling pathway were measured by RT-qPCR.

RNA-seq analyses were performed to evaluate the transcriptome changes in HGC-27 cells in response to 24 h exposure of 0.24 μM DSF with or without 0.2 μM Cu. More than 19,000 genes were screened. Compared with vehicle treated cells, 445 genes were up-regulated and 99 genes were down-regulated in cells exposed to DSF/Cu, whereas the numbers were 82 and 55 respectively in cells treated with DSF alone (Figure 2a, b). Kyoto encyclopedia of genes and genomes (KEGG) analysis showed that the MAPK signaling pathway might be involved in DSF/Cu-induced cytotoxicity (Figure 2c).

Next, we verified the data of RNA-seq via qRT-PCR analysis. The mRNAs of eight MAPK-related genes (FOS, JUN, GADD45A, CACANA1, DUSP2, HSPA1A, HSPA6, DDIT3) were significantly increased in response to DSF/Cu (Figure 2d). Additionally, it has been reported that the expression of ROS is related to the activation of MAPK pathways [31]. Next, we investigated the ROS level in DSF/Cu group.

### 3.3 DSF/Cu increased intracellular ROS level

**Figure 3.**
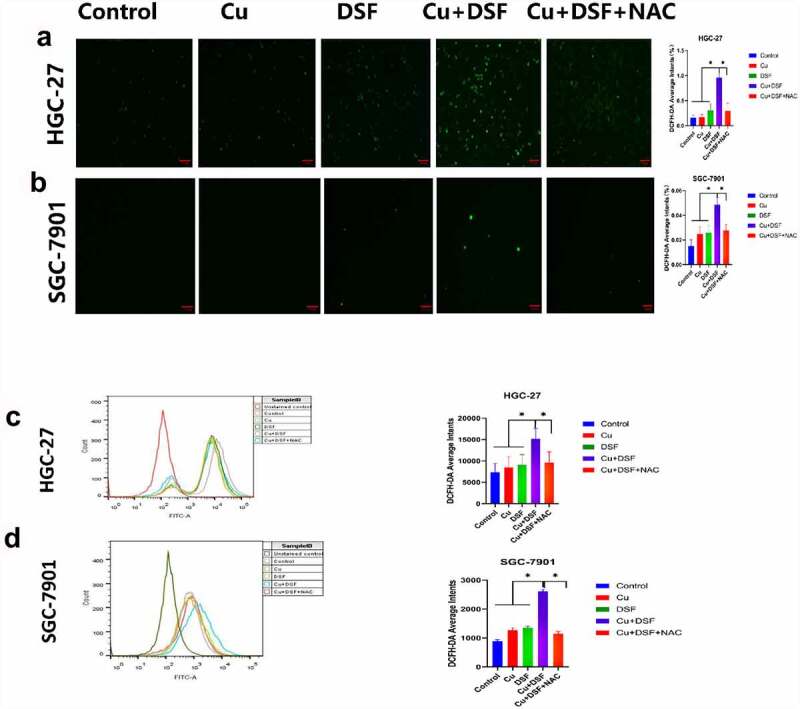
**DSF/Cu increases intracellular ROS levels**. HGC-27 and SGC-7901 cells were exposed to Cu (0.2 μM) DSF (0.24 μM or 0.3 μM), or DSF + Cu (0.24 μM, 0.3 μM + 0.2 μM) for 2 h. (a, b) ROS production was measured using fluorescence microscopy. Scale bar: 100 µm. (c, d) ROS production was measured using flow cytometry. Scale bar: 100 µm. Data are expressed as mean ± standard deviation of three independent experiments. *p < 0.05 vs. the DSF/Cu group.

After 2 h of drug exposure, the ROS level was measured and photographed using DCFH-DA probe and flow cytometry, finally results indicated the mean fluorescence intensity was higher and ROS level lowered by addition of ROS scavenger N-Acetyl-L-cysteine (NAC) in DSF/Cu as shown in Figure 3. The elevated ROS level may be the explanation of why MAPK pathway was activated.

### 3.4 The apoptosis of GC can be partly reversed by NAC *scavenger*

**Figure 4.**
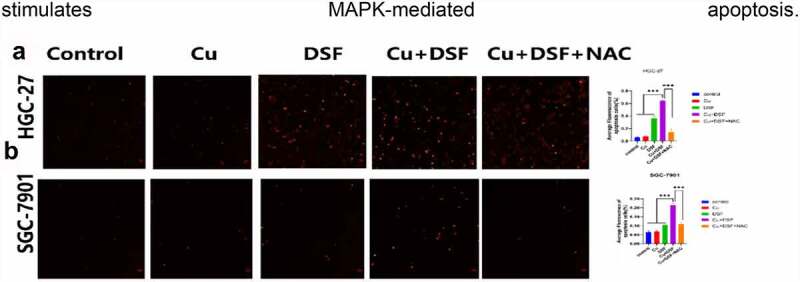
**The apoptosis of GC is partly reversed by NAC**. HGC-27 and SGC-7901 were exposed to Cu (0.2 μM), DSF (0.24 μM, 0.3 μM), or DSF + Cu (0.24 μM, 0.3 μM + 0.2 μM) for 24 h. NAC was pretreated for 12 h. (a, b) Apoptotic cells were photographed using fluorescence microscopy. Scale bar: 100 µm. Data are expressed as mean ± standard deviation of three independent experiments. * p < 0.05 vs. the DSF/Cu group.

ROS generation is associated with MAPK-mediated apoptosis. To verify MAPK-mediated apoptosis in KEGG, a TUNEL assay was performed. After 24 h of drug exposure, DSF and DSF/Cu separately induced apoptosis (Figure 4). When simultaneously exposed to DSF/Cu, apoptosis increased drastically. NAC, a ROS scavenger, diminished the increase of ROS-related apoptosis. These results suggest that the overloading of ROS stimulates MAPK-mediated apoptosis.

### 3.5 DSF/Cu increased K48-ubiquitinated proteins expression and induced NPL4 aggregation

**Figure 5.**
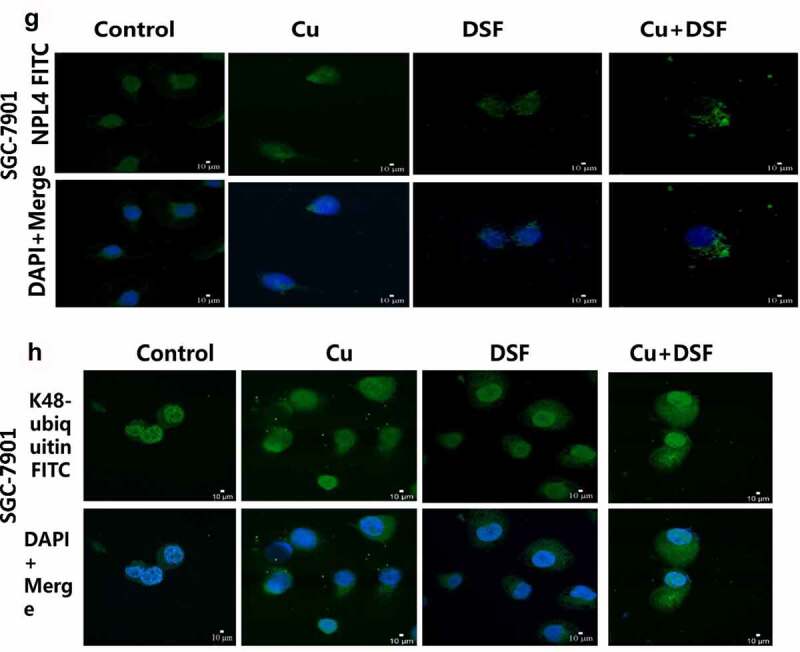
Continued.

When NPL4 aggregates, it cannot transport K48-ubiquitinated proteins to proteasomes for degradation. The expression of K48-ubiquitinated proteins was upregulated and increased in the cytosol (Figure 5a, c, f, h). NPL4 levels were repressed when complexing DSF with Cu (Figure 5b, Figure 5D). DSF and DSF/Cu led to the formation of insoluble aggregated endogenous NPL4 resistant to pre-extraction, and aggregation was evident in DSF/Cu-treated cells (Figure 5e, f).
**Figure 5.**
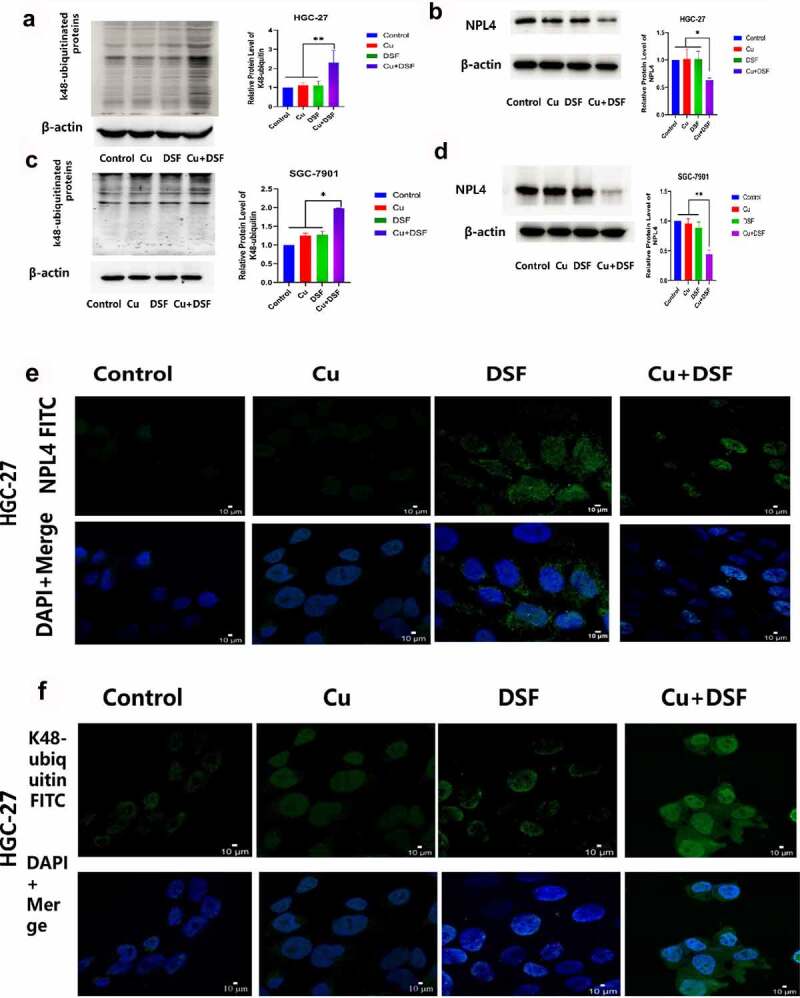
**DSF/Cu increases K48-ubiquitinated protein accumulation and induces NPL4 aggregation**. HGC-27 and SGC-7901 were exposed to Cu (0.2 μM), DSF (0.24 μM, 0.3 μM), and DSF + Cu (0.24 μM, 0.3 μM + 0.2 μM) for 24 h. (a–d) Expression levels of K48-ubiquitinated protein with NPL4.β-actin as the loading control. (e–h) DSF/Cu induces rapid cytoplasmic accumulation of K48-ubiquitinated proteins. NPL4 aggregation was visualized using immunofluorescence staining after pre-extraction. Scale bar: 10 µm. Data are expressed as mean ± standard of three independent experiments. *p < 0.05 vs. the DSF/Cu group.

### 3.6 Prognostic values of NPL4, UFD1, VCP in the whole group of patients with GC

**Figure 6.**
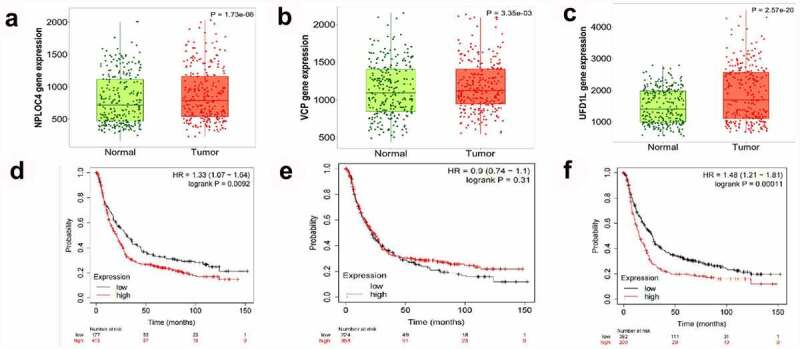
**The prognostic value of VCP and NPL4 mRNA expression using the KM plotter database**. (a, c). The expression of NPL4 and UFD1 in paired tumor and adjacent normal tissues in all GC patients. (b, d). Survival curves of NPL4 (Affymetrix IDs: 217796_s_at), UFD1 (Affymetrix IDs: 209103_s_at), and VCP (Affymetrix IDs: 208648_s_at) for all GC patients (n = 592). Red: high expression level; black: low expression level. GC, gastric cancer; HR: hazard ratio; KM, Kaplan-Meier.

The prognostic values of NPL4,UFD1,VCP mRNA expression in the whole group of patients with GC from KM plotter were collected as Figure 6. The expression of NPL4,UFD1,VCP were higher in paired tumor than adjacent normal tissues(*p*< 0.001).Furthermore, lower expression of NPL4 and UFD1 were significantly associated with a longer OS prognosis when VCP expression could not cause a significant OS difference. (Figure 6d, e, f).

## 4. Discussion

The antitumor efficacy of DSF depends on the presence or absence of Cu ions, as demonstrated in several studies [10,12,21,26,29,32,33]. Some studies showed that DSF metabolites chelate Cu ions to form CUET *in vivo and in vitro* [14,34]. Compared with DSF metabolites alone, additional Cu ions significantly promoted the formation of CUET [14,34,35]. Our cell viability and cell morphology findings suggest that DSF has cell-killing and tumor proliferation-inhibition properties, and killing is enhanced in the presence of Cu. Using KEGG analysis, we found that the MAPK pathway was more significantly altered, and more genes were upregulated in DSF/Cu-treated cells than in those treated with DSF alone. We believe that this finding might be explained because the number of Cu ions in medium culture is insufficient to form CUET and achieve the killing concentration.

ROS is associated with tumorigenesis [36] and is a by-product of normal cell metabolism. ROS activates signal transduction pathways necessary for cell growth and alters the oxidation-reduction-sensitive transcription factor NF-κβ, among others [37]. However, when ROS levels are excessively elevated, the antioxidant capacity of cells is insufficient to defend cells from oxidative stress. In these conditions, cell structures are destroyed, resulting in ROS-related apoptosis [38] that depends on the pro-apoptotic MAPK pathway [10].

In the present study, we proposed using KEGG and bioinformatic analysis to identify possible mechanisms fueling anticancer processes. We found that DSF/Cu increased intracellular ROS levels in GC cells and activated the apoptosis-related MAPK pathway. Similar to our studies, researchers elucidated the role of DSF/Cu in elevating ROS levels in head and neck squamous cell carcinoma [28], oral squamous cell carcinoma [39], and NSCLC [10]. The oxidative stress scavenger NAC partly reversed apoptosis, suggesting that oxidative stress plays a critical role in the apoptosis of GC cells.

As cofactors of VCP/p97, NPL4 and UFD1 are essential in DNA replication initiation [40] and protein degradation [14]. A recent study reported that CUET stimulates DNA replication stress, silencing the ATR pathway via immobilizing NPL4^18^. Another study found that DSF does not directly inhibit the proteasome but rather disturbs ubiquitinated protein turnover and stimulates endoplasmic reticulum stress, causing heat shock death by binding with the proteasome substrate adapter NPL4^14^. Our Western blot and confocal microscopy experiments showed that ubiquitinated proteins accumulated more in DSF/Cu-treated cells, and NPL4 aggregated with increased intensity and decreased protein levels due to immobilization by DSF/Cu. Previous studies showed that intracellular oxidative stress levels increase when ubiquitinated proteins accumulation increases and induces apoptosis [41]. Our findings suggest that the ubiquitin-proteasome system may be an essential anticancer target of DSF associated with ROS-induced apoptosis.

Based on the KM plotter, we analyzed the prognostic value of NPL4, UFD1, and VCP and found that higher expression of NPL4 and UFD1 indicated worse outcomes. Preclinical research on renal [15] and bladder cancer [42] implicated NPL4 as a target of DSF. Based on these results, we speculate that malfunction of NPL4 or UFD1 as cofactors of VCP/P97 could be the indication of DSF treatment clinically for cancer drug development.

In addition to the ROS/MAPK and NPL4 pathways, ALDH and NF-κβ are also deeply involved in the apoptotic cascade. DSF/Cu may modulate stem cells and inhibit tumor recurrence by targeting ALDH [43]. Other investigators found that DSF/Cu inhibits cell growth and proliferation of ALDH-positive breast cancer by lowering expression of ALDH [31]; the specific mechanism may involve activating ROS generation and then blockading downstream cytokine NF-κβ[43]. The latter is an ROS-induced transcription factor with the vigorous anti-apoptotic activity that reduces ROS’s pro-apoptotic effect. The inhibition of NF-κB activation enhances ROS-induced cytotoxicity, leading to apoptosis [44].

Studies indicated that DSF/Cu inhibits the growth of GC cells by modulating the stress response and Wnt/β-catenin/NF-κβ signaling [26,27]. Consistent with our study, these studies found that DSF/Cu induced anti-GC activity through the joint action of several pathways. In the present study, we used genomic analysis with RNA-seq to examine the effects of DSF/Cu on GC.

## 5. Conclusions

DSF promotes intracellular oxidative stress and induces apoptosis of GC cells via the ROS/MAPK and NPL4 pathways in a Cu-dependent manner.

## Supplementary Material

Supplemental Material
