# Supplementary material for: Disulfiram/Copper induces antitumor activity against gastric cancer via the ROS/MAPK and NPL4 pathways
Source: Bioengineered. 2022 Mar 15;13(3):6579–89. doi: 10.1080/21655979.2022.2038434 (PMC9278967; doi:10.1080/21655979.2022.2038434)
Supplement: Supplemental Material [file KBIE_A_2038434_SM4378.docx]

**Table S1. The sequences of primers for the for quantitative real-time polymerase chain reaction analysis**

| **Gene** |  | **Primer sequence** |
| --- | --- | --- |
| DDIT3 |  | F:5’-CTGCTTCTCTGGCTTGGCTGAC-3’ |
|  |  | R:5’-CTCCCTTGGTCTTCCTCCTCTTCC-3’ |
| HSPA6 |  | F:5’-CTAATGGCATCCTGAGCGTGACAG-3’ |
|  |  | R:5’-CCATCCTCTCCACCTCCTCCTTG-3’ |
| HSPA1A |  | F:5’-AAGAACGCCCTGGAGTCCTACG-3’ |
|  |  | R:5’-CTTGTCCGCCTCGCTGATCTTG-3’ |
| CACNA1C |  | F:5’-AGTCTCCACCCGCCACCAAG-3’ |
|  |  | R:5’-GCTCCTCCTCATCCTCTTCTCCTG-3’ |
| DUSP2 |  | F:5’-CTTTCCCTGCTGATCGTGCTCTTC-3’ |
|  |  | R:5’-GGCTGGTTTTGTCCCCTGTTGG-3’ |
| GADD45A |  | F:5’-AAGCGGCCAAGCTGCTCAAC-3’ |
|  |  | R:5’-ACATCTCTGTCGTCGTCCTCGTC-3’ |
| JUN |  | F:5’-AAGAACTCGGACCTCCTCACCTC-3’ |
|  |  | R:5’-GCCCGTTGCTGGACTGGATTATC-3’ |
| FOS |  | F:5’-TGCACTGCTTACACGTCTTCCTTC-3’ |
|  |  | R:5’-TCATTGCTGCTGCTGCCCTTG-3’ |
